# Analysis and review of trichomes in plants

**DOI:** 10.1186/s12870-021-02840-x

**Published:** 2021-02-01

**Authors:** Xiaojing Wang, Chao Shen, Pinghong Meng, Guofei Tan, Litang Lv

**Affiliations:** 1 Key laboratory of Plant Resource Conservation and Germplasm Innovation in Mountainous Region (Ministry of Education), Guizhou University, Guiyang, Guizhou People’s Republic of China; 2 Institute of Horticulture, Guizhou Province Academy of Agricultural Sciences, Guiyang, Guizhou People’s Republic of China

**Keywords:** Trichome, Transcription factors, Environment, Hormones, Non-coding RNA

## Abstract

**Background:**

Trichomes play a key role in the development of plants and exist in a wide variety of species.

**Results:**

In this paper, it was reviewed that the structure and morphology characteristics of trichomes, alongside the biological functions and classical regulatory mechanisms of trichome development in plants. The environment factors, hormones, transcription factor, non-coding RNA, etc., play important roles in regulating the initialization, branching, growth, and development of trichomes. In addition, it was further investigated the atypical regulation mechanism in a non-model plant, found that regulating the growth and development of tea (*Camellia sinensis*) trichome is mainly affected by hormones and the novel regulation factors.

**Conclusions:**

This review further displayed the complex and differential regulatory networks in trichome initiation and development, provided a reference for basic and applied research on trichomes in plants.

## Background

Trichomes are defined as unicellular or multicellular appendages, which are an extension of the above-ground epidermal cells in plants [1]. These appendages play a key role in the development of plantsand occur in a wide variety of species [2]. Trichomes are a protective barrier against natural hazards, such as herbivores, ultraviolet (UV) irradiation, pathogen attacks, excessive transpiration, seed spread, and seed protection. The unicellular non-glandular trichomes of *Arabidopsis* serve as an excellent model to study the molecular mechanism of trichome development in plants [3–7]. Despite the small size of trichomes, they have a marked effect on plants and human health. Cotton seed trichomes are important raw materials in the textile industry [8–10], and tea trichomes are critical for tea breeding and tea quality as they are rich in nutrients [11]. The light-leaf and light-shell phenotypes of crop rice have missing trichomes, which are conducive to crop harvesting and subsequent processing; however, the leaf trichomes in *Populus* and *Platanus* are harmful to humans, easily causing respiratory tract infections, lung infections, fever, influenza, and in severe cases, potentially cause cancer [12].

## Structure and morphology characteristics of trichomes

Trichomes are widely distributed on the surface of different organs/tissues in different plants, exhibiting various morphologies. Trichomes are generally divided into single-celled or multicellular, branched or unbranched, and glandular or non-glandular based on different characteristics and functions. Trichomes also have different shapes, such as head, star, hook and scale. Theobald and Barthlott further divided trichomes into three categories based on the distributions of leaves: large, small and glandular trichomes [13, 14]. Large trichomes are mainly distributed on the abaxial surface and margins and in the vascular bundles; small trichomes in the stomatal para cellular; and glandular trichomes are usually regularly distributed in all or part of the subepidermal tissue on the leaf surface [2]. The morphology of trichomes is associated with the spatial distribution of plant organs. The base of the tomato (*Lycopersicon esculentum*) stem is well-covered with long trichomes, whereas the upper parts of the stem has short and sparse trichomes [15]. Moreover, the density of trichomes varies in different organs. The density of trichomes on the leaf blade is significantly higher than that on the back of the blade [16]. Brewer found that different types of leaves had different densities of trichomes. In soybean, the adaxial surface of the leaf had a greater trichome density than the abaxial surface [17].

## Biological functions of plant trichomes

Plants have evolved many defense mechanisms to protect against different abiotic and biotic stresses. The morphology and density of trichomes influence several aspects of plant physiology and ecology by mediating the interactions between the plant and its environment [18]. Trichomes, along with the stomata, cutin and wax on the epidermis, performed various protective functions through synthesizing, storing and secreting many important substances [19–21]. The cotton petal trichomes maintain the shape of the buds and ensure the production of seeds [22]. In addition, trichomes protect plants from herbivores, insects and pathogens by secreting repellents, alkaloids and toxic substances [21, 23–25]. Kim et al. (2011) examined the relationship between pepper trichomes and pepper mottle virus (PepMov) resistance and showed that the resistance to PepMoV-SNU1 strain is inherited by cross combinations among pepper cultivars CM334, Chilsungcho and ECW123R [15]. In rice, two TRICHOME BIREFRINGENCE (TBR)-like proteins play an essential role in the resistance to leaf spot disease [26].

Trichomes are also important in the response to abiotic stress [22]. The presence of trichomes increases the thickness of the epidermis, and the content of long-chain fatty acids is significantly higher than that in other epidermal cells, which is helpful to reduce evaporation and regulate temperature [20, 27]. In Brazil’s high-altitude rocky areas, the plants of *Croton tiglium* and *Vriesea* effectively absorb moisture and nutrients from the atmosphere through trichomes to improve water and fertilizer utilization [28, 29]. The high-density multi-branched *Acanthophyllum squarrosum* trichomes not only have high resistance to sand burial, but also reduce mechanical damage by wind and sand [30]. In addition, the trichomes of an aquatic plant, *Salvinia molesta*, play a hydrophobic role in maintaining normal respiration [14]. Trichomes also function in signal transmission. The cell wall of *Arabidopsis* trichomes gradually thins from the top to bottom, and this change makes the base of the trichomes extremely sensitive to external stimuli. The stimulation is transmitted to cells around the base of trichomes through changes in Ca ^2+^ content and pH, thereby regulating the synthesis of defensive substances [31, 32].

## Regulatory mechanism of trichome development in plants

The development of plant trichomes is coordinated and regulated by a variety of factors, such as the environment, hormones, regulatory genes, and non-coding RNA. Among them, regulatory genes, including transcription factors and functional genes, play important roles in regulation of the initialization, growth and development of trichomes.

### Regulatory mechanism of coding genes in trichome development

**Fig. 1:**
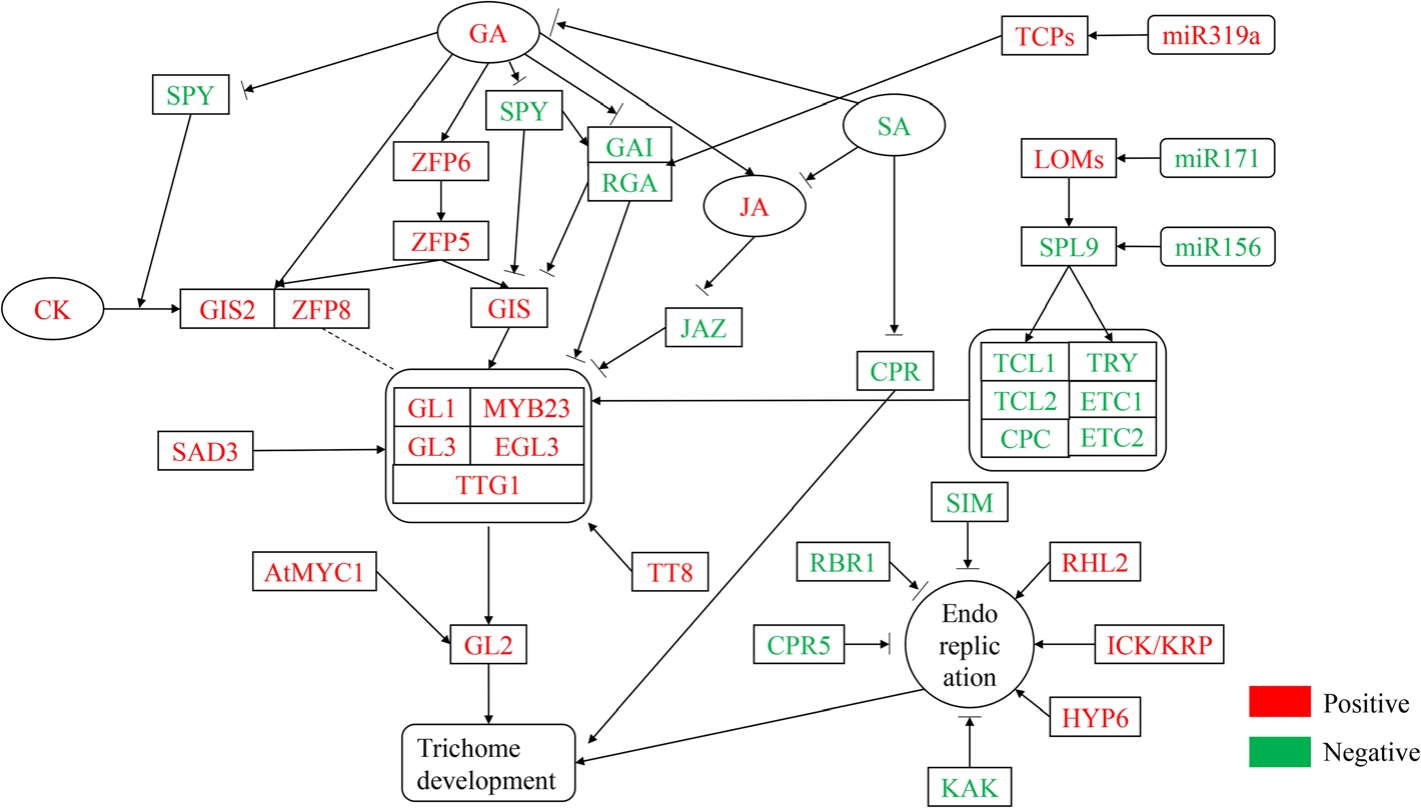
Regulatory network during trichome development. Red one represents positive regulation factor, green one represents negative regulation factor. The arrow headed lines indicate the up-regulation, and the blunted lines indicate the downregulation or inhibition

The development of plant trichomes are regulated by a complex molecular network, phytohormones, and environmental factors (Fig. 1). Previous studies have shown that many transcription factors are involved in regulating the initiation, growth and development of plant trichomes (Table 1). Positive regulatory factors include a WD40 family protein TTG1 (TRANSPARENT TESTA GLABRA1), four bHLH-like transcription factors GL3 (GLABRA3), EGL3 (ENHANCER OF GLABRA3), TT8 (TRANSPARENT TESTA), MYC-1, and three R2R3 MYB-related transcription factors (GL1, MYB23 and MYB5) [33, 41, 46, 47, 65–69]. These genes are functionally redundant and form an MYB-bHLH-TTG complex to bind to the promoter of *GLABRA2* (*GL2*) [70]. TTG1 plays a vital role in regulating the development of trichomes. Bloomer et al. (2012) found that the regulatory roles of MYB23, GL1, GL2 and GL3 in trichome development require the participation of TTG1 [71]. Complete function mutations in GL1 and TTG1 result in a loss of trichome initiation. The *gl3/egl3* double mutants also show a glabrous phenotype [72]. SAD2 (SENSITIVE TO ABA AND DROUGHT 2), an importin β-like protein, regulates trichome initiation through mediating GL3 function. In *sad2* mutants, the transcript levels of MYB23, GL1, GL2 and TTG1 were increased with a decrease in the expression of GL3 and EGL3 [73].

**Table 1:** Important genes related to trichome development in plants

Gene	Regulation	Functional description	References
*GL1*	Positive; Negative	Regulate initiation of trichome, the mutant results in trichome defect, but overexpression leads to a reduction in trichome.	[33, 34]
*TTG1*	Positive	Regulate the initiation of trichome, the mutant results in trichome defect.	[35, 36]
*GL3*	Positive	Regulates the initiation of trichome, and the mutant results in fewer trichomes.	[37, 38]
*GL2*	Positive	Regulates epidermal cell fate, the mutant results in fewer trichomes, and most of them are not branched.	[39]
*EGL3*	Positive	Function redundant with *GL3*, and *gl3/egl3* double mutants show no trichome.	[40]
*MYB23*	Positive; Negative	*myb23* single mutants exhibit reduced trichome branching but no obvious effect in trichome initiation and overexpression of the *MYB23* gene causes ectopic trichome initiation. *MYB23* has functional redundancy with *GL1*.	[41]
*TRY*	Negative	Trichome of mutant grows in clusters and branches increase.	[42]
*CPC*	Negative	The mutant exhibits reduced number of trichome and clustered trichome.	[43]
*ETC1*	Negative	*ETC1* overexpression causes a reduction in trichome formation, *etc1* single mutant has no significant phenotype. Function of *ETC1* is partially redundant with *TRY* and *CPC*.	[44]
*ETC2*	Negative	*ETC2* overexpression results in the suppression of trichomes, *etc2* single mutant shows an increase in trichome number on leaves and petioles. *ETC2* acts redundantly with *TRY* and *CPC* in trichome patterning.	[44]
*TT8*	Positive	Particularly important for marginal trichome development, no trichomes could be detected at the margin of young developing *tt8* leaves in the absence of exogenously added hormones.	[45]
*MYC-1*	Positive	*MYC1* is a positive regulator of *GL2*, the mutant with defect in trichome, and root hair pattern formation.	[46]
*MYB5*	Positive	Constitutive expression of *MYB5* resulted in the formation of smaller trichomes and ectopic trichomes and a reduction in total leaf trichome numbers and branching, the mutant displayed minimal changes in trichome morphology.	[47]
*GIS*	Positive	Regulates the differentiation of epidermal cells and inflorescences, and the trichome of the mutant is reduced.	[48]
*RHL2*	Positive	Regulate trichome nucleus replication, regulate trichome morphology.	[49]
*ICK/KRP*	Positive	Regulate trichome nucleus replication.	[50]
*HYP6*	Positive	The mutant has a small number of trichome branches and intranuclear replication (8C).	[51]
*ZWI*	Positive	The mutant has a small number of trichome branches.	[52]
*SAD2*	Positive	Regulate the initiation of trichome, mutant *sad2* presents a phenotype with reduced trichome.	[53]
*SIM*	Negative	Inhibits mitotic cycle, its mutants exhibit multicellular trichome, while other morphologies are normal.	[54]
*KAK*	Negative	The mutation of *KAK* resulted in excessively branched trichome and the nuclear DNA content reaches 64C.	[55]
*SPY*	Negative	SPY is a gibberellin signaling repressor and the *SPY* deletion mutant has increased trichome branching.	[4, 7]
*CPR5*	Negative	The *cpr5* mutant has a small number of trichome branches, the trichome volume decreases, and the intranuclear replication cycle stays at the end of the second cycle.	[56]
*RBR1*	Negative	Through its interaction with members of the E2F family of transcription factors, regulates the balance between cell division and the endocycle, and the conditional inactivation of RBR1 results in trichomes with altered morphologies, which include more branches.	[57]
*HDG11*	Negative	*HDG11* plays negative regulatory roles in trichome branching and *hdg11* mutants result in excess branching of trichomes.	[58]
*GLH*	Positive	*GLH* can induce the formation of papillae in the late stage of development, *glh* mutants showed defects in papillae formation and reduces in cellulose content.	[59]
*TBR*	Positive	*TBR* is required for secondary wall cellulose synthesis, the *Arabidopsis tbr* has severely reduced crystalline cellulose in trichomes and reduced trichome density, altered trichome shape and surface appearance, lack of trichome papillae and basal cells, altered stomata shape, and altered patterns of callose deposition.	[60]
*NOK*	Positive	The *noeck* (*nok*) mutant has trichomes with increased branching and a glassy transparent appearance but displays no increase in nuclear DNA content.	[61]
*MIXTA*	Positive	Overexpression of *MIXTA* led to the growth of a large number of trichomes on cotyledons, leaves, and stems in tobacco.	[62]
*CotMYB*	Positive	Overexpression of *CotMYB* does not change its phenotype in *Arabidopsis* but increases the number of tobacco trichomes.	[62]
*GaMYB2*	Positive	Cotton fibrin *GaMYB2* not only regulates the leaf and stem trichome development of the *Arabidopsis gl1* mutant, but also induces the formation of seed trichome.	[63]
*AnnGh3*	Positive	Overexpression of *AnnGh3* isolated from cotton in *Arabidopsis* resulted in a significant increase in leaf trichome density and length.	[64]

Negative regulators include CAPRICE (CPC), TRIPTYCHON (TRY), ENHANCER OF TRY AND CPC1 (ETC1), and ETC2, which show functional redundancy. Negative regulators that interact with GL3, EGL3, and TTG1 negatively regulate the development of trichomes [73]. In addition, ETC1 and ETC2 act as enhancers of TRY and CPC to promote the movement of TRY between cells and inhibit the initiation of trichome development by interacting with GL3 and TTG1. CPC and TRY have similar functions [42].

Presently, two models have been proposed for the molecular regulatory mechanism of trichome development that co-regulate the growth and development of trichomes in plants. The first is the activator-depletion model: the TTG1 has a dual role in trichome development, moves freely between cells and combines the GL3/EGL3 and GL1/MYB23 to form a GL1/MYB23-GL3/EGL3-TTG1 complex that positively regulates trichome development [74]. The second is the activator-inhibitor model: the MYB-bHLH-TTG complex activates the TRY/CPC and promotes TRY and CPC proteins to move into adjacent cells. This complex forms an inactive TRY/CPC-GL3/EGL3-TTG1 complex and negatively regulates trichome formation by replacing the positively regulated transcription factor GL1/MYB23 [75]. SAD2 maintains the stability of the GL1-GL3-TTG1 complex by regulating the accumulation of mRNAs of many trichome-related genes in plants, which in turn affects protein activity [53].

In addition, some regulatory factors affect trichome branching by modulating the nuclear replication of trichome cells. The formation of trichome branches occurs after four times of endoreplication of trichome nuclear DNA. Among them, the positive regulators affecting the DNA replication and branching processes are INTERACTORs of CDK/KIP-related protein (ICK/KRP), ROOT HAIRLESS 2 (RHL2), HYPOCO-TYL 6 (HYP6), ZWICHEL (ZWI), GLABROUS INFLORESCENCE STEMS (GIS), GL3 and TTG1.

Misexpression of the ICK1/KRP1 in single-celled *Arabidopsis* trichomes reduces endoreduplication, cell size, and induces cell death [50]. The *zwi, rhl2* and *hyp6* mutations reduce trichome branching [76]; AtFIP37 and GIS positively regulate plant trichome branching through an intranuclear replication cycle [77, 78]. Negative regulators of trichome branching include SIAMESE (SIM), KAKTUS (KAK), SPINDLY (SPY), CONSTITUTIVE PATHOGEN RESPONSE 5 (CPR5), RETINOBLASTOMA RELATED 1 (RBR), TRY, and HOMEODOMAIN GLABROUS 11 (HDG11). SIM, CPR5, and RBR all affect trichome branching by controlling nuclear replication [42, 56, 66]; KAK mainly regulates the expression pattern of GL3 and EGL3 causing reduced trichome branching [55]; SPY is a negative regulator controlled by gibberellin [79], and HDG11 can restore the phenotype of the *gl2* mutant in the *Arabidopsis* trichome [58].

The development of trichome and plant organogenesis occurs synchronously. GLASSY HAIR (GLH), TBR, NOECK (NOK), and their protein complexes together induce the formation of trichomes. The mutant phenotypes of the three genes were all glassy trichomes [59–61]. The GLH can induce the formation of papillary hairs in the late development of trichomes, and the cellulose content of *glh* mutants was decreased in *Arabidopsis* [59]. In addition, the complex GL1-GL3-TTG1 affects the signal transmission between cells by activating negative regulatory genes of trichome development, such as CPC, TRY and ETC1, resulting in the inhibition of the activity of the GL1-GL3-TTG1 complex and promotion of the development of cells into non-trichome cells [50].

However, the regulatory mechanism of trichome development is different in various plants. Overexpression of *Arabidopsis* GL1 in tobacco (*Nicotiana tabacum* L) does not affect trichome development. Overexpression of the snapdragon MIXTA and CotMYB (a MIXTA homologous gene in cotton) genes increases the density of *N. tabacum* trichome, while transgenic *Arabidopsis* expressing the snapdragon MIXTA shows no change [68]. In addition, overexpression of the cotton TRY hindered the development of tobacco trichomes, while transgenic *Arabidopsis* has no change [64]. *Arabidopsis* trichome suppressor CPC has no effect on *L. esculentum* trichome traits [80]. Control of trichome initiation GL1 and trichome differentiation OCL4B (OUTER CELL LAYER 4) inhibition of maize [81] regulated the growth and development of the trichome only in *Arabidopsis* not in *N. tabacum, P. hybrida* L*.*, and *L esculentum* [82]. Thus, all studies showed that there are different regulatory mechanisms during multicellular and unicellular trichome development. Nevertheless, there is a specific relationship between them. For example, the cotton trichome transcription factor GaMYB2 can restore the trichome phenotype of the *gl1* mutant in *Arabidopsis* [63], and the protein AnnGh3, when isolated from cotton, promotes the trichome elongation of *Arabidopsis* [64].

Other transcription factors related to multicellular trichomes have also been studied. WO (WOLLY) affects the initial development of *L. esculentum* trichome [83]. Cucumber CsGL1 controls the development of trichome branch [84]. Moreover, transposable elements are involved in cotton fiber cell development [85]. m6A (methylations at position N6 of internal adenosines) residues are directly accommodated by its YTH reader. The ECT2 protein containing the YTH domain decodes the m6A signal during development and regulates trichome branching by controlling their ploidy levels [86, 87]. *SlCycB2* overexpression results in *L. esculentum* plants with no non-glandular and glandular trichomes [88].

### Regulation of non-coding genes

MicroRNAs (miRNAs) are endogenous non-coding small RNAs approximately 21 nucleotides in length with high complementarity sequences to their target mRNAs. These miRNAs control many aspects of cellular functions by modulating the expression level of their target genes at the post-transcriptional level [89, 90]. miRNAs regulate trichome development by modulating the expression of SQUAMOSA PROMOTER BINDING PROTEIN LIKE (SPL) [91]. miR156 is a potential graft-transmissible miRNA that modulates leaf morphology and produces long leaf trichomes [92]. In *Arabidopsis*, SPLs are negative regulators of trichome development in the inflorescence stem and floral organs. The *Arabidopsis* SPL gene family contains 17 members, 10 of which are targeted by miR156 [93]. The expression of miR156 and SPLs (SPL3/SPL*9*) are temporally regulated [94, 95]. The expression level of miR156 is the highest in young plants and declines as the plant ages, while the expression level of their targets, SPLs, are low in young plants and accumulates gradually during reproductive growth. The expression pattern was consistent with the gradual loss of trichomes on the stem and floral organs. Overexpression of a mimicry target of miR156 significantly decreased the density of stem trichomes. Similar phenotypes were found in transgenic plants overexpressing miR156-resistant forms of SPL3, SPL9 and SPL10. The SPL9, binding to the promoters of TCL1 and TRY, activates their expression and inhibits the formation of trichomes [91]. In addition, transgenic *Arabidopsis* constitutively overexpresses miR156 producing ectopic trichomes on the stem and floral organs. Transgenic alfalfa (*Medicago sativa*) overexpressing miR156 showed increased trichome density, delayed flowering time, and elevated biomass production owing to decrease the expression level of three target *SPLs* (SPL6, SPL12 and SPL13). We found that the miR171-targeted LOST MERISTEMS 1 (LOM1), LOM2 and LOM3 were also involved in regulating SPL activity. Reduced LOM abundance by overexpression of miR171 leads to decreased trichome density on the stems and floral organs. Conversely, constitutive expression of the miR171-resistant LOM (rLOM) promotes trichome production [96]. The recent study had revealed that the expression level of TCP, as a target of miR319a, was decreased in miR319a-Ox transgenic poplars and significantly increased the density of leaf trichomes. On the contrary, the use of short tandem target mimics (STTM) to repress miR319a, the transcription level of TCP was upregulated and decreased the trichome density (Table 2) [97].

**Table 2:** MicroRNAs related to trichome development in plants

MicroRNAs	Regulation	Functional description	References
miR156	Negative	miR156-targeted SPL transcription factors, overexpression of a mimicry target of miR156 significantly increases the SPLs transcripts and decreases the density of stem trichomes.	[95]
miR171	Negative	miR171-targeted LOM1, LOM2, and LOM3 regulating the SPL activity. Overexpression of miR171 led to decreased trichome density on stems and floral organs.	[96]
miR319a	Positive	miR319a-targeted TCPs, overexpression of miR319a decreased transcription levels of TCPs and significantly elevated leaf trichome density in transgenic poplar.	[97]

### Role of hormones in growth and development of trichomes

The differentiation of trichomes is closely related to its location, growth, development stage, and hormone level (Table 3). Studies have shown that gibberellin (GA) [101], cytokinin (CK) [102], salicylic acid (SA) [98], jasmonic acid (JA) [103], brassinolide (BR), and other hormones regulate the initiation, growth and development of plant trichomes. GA treatment increases trichome numbers on *Arabidopsis* leaves. GA enhances the expression levels of MYB23, GL1, GL3 and EGL3, while inhibiting the expression levels of TRY, ETC1 and ETC2 [74]. Analysis of *Arabidopsis ga1-3, spy* mutants and wild-type showed that an endogenous GA level and/or activity of the signal transduction pathway positively regulates the number and branching of trichomes [4, 97]. GA and CK control trichome formation primarily through C2H2 transcription factors, such as GIS1, GIS2, ZINC FINGER PROTEIN (ZFP) 5, ZFP6 and ZFP8, which act upstream of the GL3/EGL3-GL1-TTG1 transcriptional activator complex.

**Table 3:** The phytohormones related to trichome development in plants

Hormone		Functional description	References
GA	Positive	GA enhances the expression levels of MYB23, GL1, GL3, and EGL3, but inhibits the expression levels of TRY, ETC1, and ETC2, and positively regulates the number and branching of trichomes.	[72]
CK	Positive	CKs increase trichome formation, regulate C2H2 transcription factors ZFP8 and GIS2 acting downstream of SPY and upstream of GL1.	[48]
SA	Negative	SA decreases both trichome density and number.	[98]
JA	Positive	JA degrades JAZ to release WD-repeat/bHLH/MYB complex and initiate trichome.	[99]
Ethylene	Positive	Ethylene affects trichome branching, *Arabidopsis* ethylene receptor mutant, *etr2–3*, has completely unbranched trichomes.	[99]
BR	Positive	-	[100]

CKs promote trichome formation [45]. The GA response inhibitor SPY activates the CK signaling pathway in *Arabidopsis* [104]. GA and CK signaling pathways control trichome cell fate by integrating the *Arabidopsis* transcription factors GIS, ZFP8, GIS2 to regulate GL1 expression. The study revealed that ZFP8 and GIS2 are involved in the regulatory mechanism of CK on trichome production. GIS2 induced by CKs acts downstream of SPY but upstream of GL1.

SA reduces trichome formation [97]. The *Arabidopsis cpr* mutant that overproduces SA showed a low trichome density. SA reduced the positive effects of JA on trichome induction, suggesting negative crosstalk between the JA- and SA-dependent pathways. There is also evidence for interactions among GA, JA and SA pathways, and *Arabidopsis* treated with SA showed a 1/4 reduction in trichome density. In the induction of trichome formation, GA and JA act synergistically, but SA acts antagonistically to the effect of GA with respect to trichome density and numbers. Exogenous JA application significantly increased the density of trichomes in *A. annua* and *L. esculentum* [105]. The reduction of JA levels (through silencing of OPR3, a key enzyme in the biosynthesis of the precursor of JA) impairs glandular trichome development in *L. esculentum* [106]. The Jasmonate ZIM-domain (JAZ) proteins interact with bHLH transcription factors (GL3, EGL3 and TT8) and MYB transcription factors (MYB75 and GL1), which are degraded by JA to regulate the initiation and development of trichomes through releasing a WD-repeat/bHLH/MYB complex and activating downstream factors [99].

Ethylene increases trichome branching in cucumber [107]. Increasing ethylene synthesis enhances the length of trichome branches in cotton [108, 109]. The ethylene receptor ETR2 controls trichome branching by regulating the assembly of microtubules [110]. A recent study revealed that *L. esculentum* SlIAA15, acting as strong repressor of auxin-dependent transcription, is involved in regulating the initiation of trichomes [100]. Moreover, the application of BR in cotton increases the expression level of fiber-related genes in cotton ovules, while Brz (BR inhibitor) inhibits fiber-related gene expression [111].

### Effects of environmental factors on trichome development

Trichomes help plants to deal with different environmental challenges. To better adapt to the environment, trichomes have evoluted to adapt to a complex and changing environment, such as salt, temperature, light, and other abiotic factors [112–114]. In a salt stress experiment, the trichome size and density of mint and marjoram increased significantly [115, 116]. The trichome density of *Solanum habrochaites* is affected by photoperiod and temperature. Under a long day of natural sunlight, trichome density increased; the density increased at temperatures of 20-25 °C and decreased at low temperatures [117]. Ning et al. (2016) selected two experimental sites (from wet to dry) along the Loess Plateau latitude gradient to observe changes in the trichome morphology of *Caragana korshinskii* [114]. Micro-phenomena through which trichomes grew denser and larger under reduced precipitation were observed using a scanning electron microscope. Evidence that trichomes have structural adaptations to low temperatures and enhance tolerance to freezing was obtained from a study on birch where frost rapidly increased the density of glandular trichomes [118]. Yan et al. (2012) found that exposure of *Arabidopsis* to UV-B could stimulate trichome formation [119]. Plants from higher latitudes tended to be shorter, have fewer branches, flower earlier, flower larger, develop fewer trichomes, and produce more highly lobed leaves than plants at lower latitudes [120]. The surface density of *M. sativa* increases after mechanical injury, drought stress or growing under water deficit conditions [121].

## Research on trichomes in tea plants (*C. sinensis*)

The molecular mechanism of trichome formation has also been investigated in other model plants, such as *C. sativus* [122], *L. esculentum* [123], *B. campestris* [124] and *B. napus* [125]. The recent study had revealed that another regulatory mechanism might be involved in regulating trichome development in plants. Trichome (also referred to as ‘háo’ in tea) was generally regarded as one of the quality standards of tea due to secrete various secondary metabolites, such as flavonoids, tea polyphenols, and amino acids. The number of tea trichomes depends on degree of tenderness of tea leaves. The distribution of trichomes is especially thick on newly budded leaves and gradually decreases as the leaf develops [126]. Previous studies had revealed that over 20,000 differentially expressed genes (DEGs) were identified in FDDB (Fudingdabaicha, trichome-rich) and RCZ (Rongchunzao, trichome-less) [127]. However, the homologous sequences of *Arabidopsis* trichome-related genes were not significantly different between the two cultivars or was even undetected [127].

Two hypotheses were proposed to explain the above phenomenon. First, the low proportion of tea trichomes in collected leaves resulted in the relatively low transcripts of the trichome-related genes. Second, it is possible that a novel pathway has not been found about trichomes formation in tea plant. Previous study had revealed that the trichome-related genes, including four HB regulators (REVOLUTA, WOX1/WOX8, and ATHB-51), RAV1, SPLs and TCPs, were up-regulated in ‘RCZ’ tea cultivar. The expression level of *ZFP4* gene was decreased in ‘RCZ’. These results were consistent with the previous studies that these genes played a key role in regulating trichome development in *Arabidopsis* [102, 128–133]. Phytohormones also play an important role in tea trichome development. Although the genes involved in each type of phytohormone signal were differentially expressed, those related to CKs and encoding the GA receptor GID1b were repressed in RCZ; however, DELLAs, which encode the major GA signaling repressors, were up-regulated. These results indicate that both GA and CK signals are suppressed in RCZ. Moreover, the functions of GA and CK could be influenced by SPINDLY, an O-linked *N*-acetylglucosamine transferase that suppresses GA signaling and promotes CK responses [48, 109, 132, 134]. The DEG dataset showed that the expression of SPINDLY was also down-regulated. Collectively, these results suggest that the hairlessness of RCZ might result in part from the repression of GA and CK signals. Trichomes contain cellulose for the establishment of thick cell walls, which is regulated by the trichome development phase [135]. In this study, we found that most TBR-like family (TBR and TBL) and cellulose synthase genes, which are important for cellulose synthesis, were repressed in RCZ, and this repression was markedly associated with the hairless phenotype of the RCZ cultivar.

Although tea trichomes have no significant effect on the content of leaf inclusions account for their small proportion in a leaf. But trichomes really exist some trichome-synthesized flavoring materials, including catechins, flavonols, amino acids, purine alkaloids and volatiles which are important ingredient in tea flavors. More importantly, some defensive metabolites and many anti-insect, anti-microbial genes highly accumulated and expressed in the trichomes, respectively, strongly indicated that trichomes may also play an important in protecting tea plants against biotic and abiotic stresses [11, 136].

## Outlook

In recent years, although the great progress has been made in the molecular mechanism of trichome development in *Arabidopsis*, the research on the regulation mechanism of trichome development in non-model plants is still unclear. Further research is urgently needed to be explored the molecular mechanism of trichome development in non-model plants. With the development of next-generation sequencing technology, the combination of genomics and classical genetics provides a very powerful tool to identify candidate genes regulating trichome development. CRISPR/CAS9-mediated knockout and siRNA-mediated knockdown technologies was used to identify the function of candidate genes by comparing the difference between the mutant and control plants. Moreover, trichome, as a barrier for plants to resist different stresses, played an important role in coping with biotic and abiotic stresses. Through transcriptomics, proteomics combined with metabolomics were used to further clarify the complex signal conversion mechanism. The molecular mechanism of plants converts environmental stimuli into endogenous signals that in turn regulate the growth and development of trichome and affect the structure, density and function, which still unclear. On the other hand, an important feature of glandular trichome is that it specifically synthesizes a wide range of secondary metabolites. These secondary metabolites not only play an important role in response to biotic and abiotic stress, but also contribute to human production and life, having very important economic value. For example, artemisinin, a sesquiterpene lactone, is isolated from the glandular trichome of Artemisia annua and widely used for its high anti-malarial pharmacological effects [137]. The glandular trichome of *C. sativa* can synthesize and accumulate cannabi-noids, which are prenylated polyketides having important pharmacological effects in clinical studies [138]. Modern genetic engineering techniques are used to transform plant glandular trichome into ‘chemical factories’ to synthesize valuable compounds for humans also has great development prospects.

## Conclusions

Trichomes have a marked effect on plants and human health, its growth and development are effected by environment factors, hormones, transcription factor, non-coding RNA, etc. Although several previous studies had summarized the factors that affected the formation and development of plant trichomes, their reviews lacked a comprehensive summary and only focused on transcription factors, hormones and environment in model plants. The aim of this review is not only to summarize the factors, such as environment factors, hormones, transcription factor, non-coding RNA, and functional genes, regulate the formation and development of plant trichomes in model plants, but also investigate the regulation mechanism of trichome development in non-model plants, such as tea. We are expected this review provided a reference for basic and applied research on trichomes in plants in the near future.

## Data Availability

Not applicable.
